# Life-threatening arrhythmia in patients with suspected acute myocarditis

**DOI:** 10.1016/j.ijcha.2025.101718

**Published:** 2025-06-19

**Authors:** Melina Krempke, Jasmin Büchel, Kseniya Bulatova, Gianmarco M. Balestra, Philip Haaf, Jeanne Pouly, Paul Drews, Christian Mueller, Sven Knecht, Patrick Badertscher, Felix Mahfoud, Michael Kühne, Christian Sticherling, Philipp Krisai

**Affiliations:** Department of Cardiology and Cardiovascular Research Institute Basel, University Hospital Basel, University of Basel, Basel, Switzerland

**Keywords:** Acute Myocarditis, Arrhythmia, Sustained ventricular tachycardia, Ventricular fibrillation, Cardiac arrest, Troponin

## Abstract

**Background:**

Patients with acute myocarditis (AM) often undergo prolonged rhythm monitoring due to the risk for life-threatening arrhythmia.

**Objective:**

To describe the occurrence, timing and potential early rule-out of life-threatening arrhythmia in patients with AM.

**Methods:**

We included consecutive patients with suspected AM admitted to the ICU/IMC for continuous rhythm monitoring into a cohort study. We assessed the incidence and timing of life-threatening arrhythmia (sustained ventricular tachycardia, ventricular fibrillation, cardiac arrest). To rule-out arrhythmia, we evaluated left ventricular ejection fraction (LVEF), maximal cardiac troponin-T (cTnT) levels and a multivariable model.

**Results:**

Among 304 patients with AM (41 ± 16.6 years, 27 % female), 13 life-threatening arrhythmias occurred in 10 (3.3 %) patients. Of these, 8 occurred within 24 h, 2 between 24–48 h and 3 after 72 h of hospitalization. Patients with life-threatening arrhythmia had substantially higher mortality rates (40 % vs. 0.3 %, p < 0.001). There was no binary cut-off for LVEF and cTnT to rule-out arrhythmia. The last life-threatening arrhythmia occurred before the cTnT-peak in 3 (42.9 %), simultaneously with the peak in 1 (14.3 %), and after the peak in 3 (42.9 %) patients. The final multivariable model included female sex, cTnT, and LVEF and demonstrated an area under the curve of 0.98 (95 % CI 0.96–1), with a sensitivity of 99 % and specificity of 75 % to rule-out life-threatening arrhythmia.

**Conclusions:**

In patients with suspected AM, life-threatening arrhythmias were rare but associated with a 40% mortality rate. A combined model including 3 clinical variables ruled-out life-threatening arrhythmia with a high sensitivity and may help to guide the indication of rhythm monitoring.

## Graphical abstract

**Figure:**
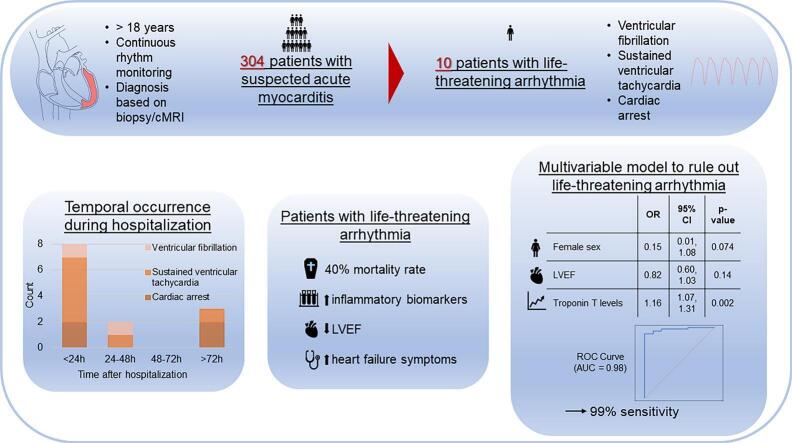

## 1 Introduction

Acute myocarditis (AM) is an acute inflammatory disease of the myocardium that carries an increased risk for life-threatening arrhythmias [[1], [2], [3]]. In the acute phase, the risk for ventricular arrhythmias ranges from 6 % to 30 %, depending on the patient population and the specific definition of arrhythmia used [[4], [5], [6], [7]]. Consequently, patients with suspected AM often undergo prolonged electrocardiography (ECG) monitoring in intensive or intermediate care units (ICU/IMC) [8], which can lead to patient discomfort and impose significant economic burden. As diagnostic procedures for AM become more sensitive – particularly with advancements in high-sensitivity cardiac Troponin T (hs-cTnT) testing and cardiac MRI – the patient population requiring monitoring is expected to grow [9]. This underscores the need for precise and timely identification of AM patients at very low risk for life-threatening arrhythmia to appropriately discharge those who do not require extended rhythm monitoring.

Currently, there is lack of evidence on the temporal occurrence and characterization of life-threatening arrhythmia in patients with suspected AM during the index hospitalization [10,11], and no guidelines exist regarding the optimal duration of monitoring [12,13]. In clinical practice, physicians often rely on cardiac imaging parameters and biomarkers to guide rhythm monitoring decisions. In particular, hs-cTnT is frequently used for risk stratification [14], with the assumption that the risk of life-threatening arrhythmia decreases after peak levels of hs-cTnT (hs-cTnT_peak_) have been reached. However, this commonly used approach has not been thoroughly investigated in prior studies. Accurate identification of patients truly at low life-threatening arrhythmia risk is crucial, as missing a life-threatening arrhythmia event could have serious consequences.

Therefore, we aimed to characterize the incidence and timing of life-threatening arrhythmia in patients with suspected AM undergoing continuous rhythm monitoring. We also aimed to evaluate the performance of commonly used approaches to rule out life-threatening arrhythmia and to build a clinical model to identify patients who may not require we only inco the ICU/IMC for rhythm monitoring.

## 2 Methods

The Basel Myocarditis Cohort study is an ongoing registry at the University Hospital Basel, Switzerland, a tertiary academic hospital. The study protocol was reviewed and approved by the local ethic committee of Northwest- and Central Switzerland (project ID 2017–01783) and was conducted in accordance with the principles of Good Clinical Practice and the Declaration of Helsinki.

### 2.1 Patient population

We included all patients ≥ 18 years with a diagnosis of suspected myocarditis and perimyocarditis who were subsequently hospitalized at an ICU/IMC with continuous rhythm monitoring between January 2000 and September 2023. We excluded patients not hospitalized at an ICU/IMC and with written refusal to participate. AM was diagnosed by a cardiologist according to current guidelines [15]. Admission to the ICU versus IMC was at the discretion of the treating physician and based on hemodynamic stability or assumed need for advanced therapy during hospitalization. Patients with mild cases who could be managed on a general ward were excluded. Patients underwent cardiac MRI (n = 296) and/or endomyocardial biopsy (EMB) (n = 22) for diagnosis. Patients with suspected AM but no cardiac MRI or EMB (n = 48) were excluded. (S1) In patients without EMB, the diagnosis of clinically suspected AM was determined by two independent cardiologists according to current recommendations[15] and based on cardiac MRI [16], additionally taking into account clinical manifestation, ECG findings, transthoracic echocardiographic imaging (TTE), laboratory markers and medical history [17].

### 2.2 Clinical, laboratory and imaging investigations

Demographic and clinical data were extracted from the electronic health record. We used the presenting 12-lead ECG with a paper speed of 25 mm/s and an amplification of 10 mm/mV. ST segment elevations greater than 1 mm, respectively greater than 2 mm in V2-V3, that were not attributable to a single coronary artery territory were considered diffuse ST elevations. Inverted T-waves not attributable to a single coronary artery territory were considered diffuse T-wave inversions. PQ depressions greater than 0.5 mm were considered significant.

For blood analysis, inflammatory biomarkers (C-reactive protein [CRP], leucocytes, lymphocytes, neutrophils, eosinophils and procalcitonin) as well as cardiac biomarkers (N-terminal pro-B-type natriuretic peptide [NT-proBNP], Creatin-Kinase-MB [CK-MB], cTnT) were assessed. According to local standards, conventional cTnT was measured until 2010, after which high-sensitivity cTnT was used. For the current analysis, we pooled conventional and hs-cTnT levels for the overall cohort but used only hs-cTnT levels to define hs-cTnT_peak_ in patients with at least 2 hs-cTnT measurements. For all other biomarkers, maximal values during hospitalization were recorded. TTEs were performed by trained cardiologists or cardiac sonographers, and analyses were conducted by board-certified cardiologists. Cardiac MRIs were performed on a 1.5 T or 3 T (MAGNETOM Avanto/Avanto fit resp. Skyra, Siemens Healthineers, Erlangen, Germany). All MRI studies followed a standard clinical procedure with ECG-triggering that included time-resolved (cine) balanced steady-state free precession (bSSFP) imaging, T1- and T2-Mapping and late gadolinium enhancement (LGE) sequences. Patients were continuously monitored during their whole stay at the ICU/IMC by a bedside patient monitor (Philips IntelliVue MX800) that automatically detected arrhythmias- Detected arrhythmias were transferred to a patient data management system (PDMS) (Metavision [V5.46.44; iMDsoft, Germany] or CareVueChart [V: Rev.D03.02; Philips Healthcare, Netherlands]), which were evaluated by trained study personnel. For the combined endpoint of life-threatening arrhythmia, we included patients with sustained ventricular tachycardia (VT) (≥30 sec), ventricular fibrillation (VF), and/or cardiac arrest (CA). Arrhythmic events occurring after transfer out of the ICU/IMC were not captured. Other cardiovascular outcomes including all-cause mortality and cardiogenic shock were captured throughout the whole hospitalization including general wards.

### 2.3 Statistical analysis

Baseline characteristics were stratified based on presence or absence of life-threatening arrhythmia. Continuous variables were reported as mean ± standard deviation or median (interquartile range [IQR]) and compared using the Mann-Whitney *U* test or the Wilcoxon rank-sum test, as appropriate.

To investigate potential predictors to rule-out life-threatening arrhythmia, we used two approaches. First, we investigated the discriminatory performance of frequently used, common variables in clinical practice for life-threatening arrhythmia. These included the absolute values of left ventricular ejection fraction (LVEF), and maximal cTnT concentration for the binary outcome of life-threatening arrhythmia yes or no, as well as the temporal association of cTnT_peak_ concentration with the occurrence of the latest life-threatening arrhythmia during monitoring. Second, we used univariable logistic regression models to investigate the following potential predictors to rule-out life-threatening arrhythmia: age, sex, LVEF, Killip Classification, cTnT, CK-MB, neutrophils, CRP and ECG findings at presentation (ST depression, ST elevation, PQ depression and QTc duration) individually. Patients with missing values for these variables were excluded from calculations of the regression models without imputation to limit potential confounders. We then selected variables for a multivariable model by checking the multicollinearity with the Variance Inflation Factor (VIF), defining a cutoff value of 5. To find the best model, we calculated the prediction error with the Akaike Information Criterion (AIC) for the absence of life-threatening arrhythmia with the stepAIC function. To determine the predictive performance of the multivariable model to rule-out life-threatening arrhythmia, we then calculated the Receiver Operating Characteristic (ROC) curve and the Area Under the Curve (AUC). In this model, we aimed to maximize the sensitivity to rule-out life-threatening arrhythmia and accepted a lower specificity in order to be able to safely rule-out low risk patients, choosing the threshold of sensitivity at 99 %. Calibration was not calculated due to low number of events. Since the positive outcome in our model corresponds to the prediction of no life-threatening arrhythmia, the positive predictive value is interpreted as the probability of no disease. This differs from the common approach of positive predictive value as the presence of disease. In sensitivity analyses for the model, we included only patients with hs-TnT measurements.

All statistical analyses were done using statistical software R version 4.3.2. A p-value ≤ 0.05 was considered statistically significant.

## 3 Results

### 3.1 Baseline characteristics

Baseline characteristics for the 304 included patients with suspected AM, overall and stratified by the occurrence of life-threatening arrhythmia, are shown in Table 1. The mean age was 41 ± 16.6 years, and 73 % were male. The most common presenting symptoms were chest pain (83 %), dyspnoea (30 %) and fever (29 %). On the presenting ECG, 29 % of patients had PQ depression, 31 % had ST-elevations and 33 % had negative T waves. The median LVEF was 56 % (IQR 50–61), and 86 % of patients exhibited LGE on cardiac MRI.

**Table 1:** Baseline characteristics in patients with acute clinically suspected myocarditis.

	**Overall**	**Life-threatening arrhythmia**		
**Characteristic**	n = 304	**no**, n = 294	**yes**, n = 10	**p-value**
Female Sex	81 (27 %)	75 (26 %)	6 (60 %)	**0.039**
Age, years	41.0 ± 16.6	40.6 ± 16.5	53.7 ± 16.4	**0.017**
Hospital stay, days	8.6 ± 11.5	8.4 ± 11.5	12.0 ± 12.1	0.44
In-hospital death	5 (1.6 %)	1 (0.3 %)	4 (40 %)	**<0.001**
Presenting symptoms and clinical signs				
Chest pain	253 (83 %)	248 (84 %)	5 (50 %)	**0.015**
Palpitations	19 (6.3 %)	16 (5.4 %)	3 (30 %)	**0.013**
Dyspnoea	90 (30 %)	83 (28 %)	7 (70 %)	**0.013**
Syncope	13 (4.3 %)	11 (3.7 %)	2 (20 %)	0.088
Fever	87 (29 %)	85 (29 %)	2 (20 %)	0.80
Respiratory infection	103 (34 %)	101 (34 %)	2 (20 %)	0.55
Peripheral edema	15 (4.9 %)	13 (4.4 %)	2 (20 %)	0.14
Pericardial friction	5 (1.6 %)	5 (1.7 %)	0 (0 %)	>0.99
Presenting with arrhythmia	11 (3.6 %)	9 (3.1 %)	2 (20 %)	0.050
Killip classification, number				**<0.001**
1	273 (90 %)	270 (92 %)	3 (30 %)	
2	11 (3.6 %)	10 (3.4 %)	1 (10 %)	
3	5 (1.6 %)	4 (1.4 %)	1 (10 %)	
4	15 (4.9 %)	10 (3.4 %)	5 (50 %)	
ECG at presentation				
PQ depression	87 (29 %)	85 (29 %)	2 (20 %)	0.80
ST elevation	93 (31 %)	90 (31 %)	3 (30 %)	>0.99
Negative T wave	99 (33 %)	92 (31 %)	7 (70 %)	**0.026**
LVEF, %	56.0 (50.0–61.0)	56.0 (50.0–61.0)	30.0 (21.8–35.0)	**<0.001**
Late gadolinium enhancement on MRI	261 (86 %)	256 (87 %)	5 (50 %)	**0.004**
Endomyocardial biopsy	22 (7.2 %)	16 (5.4 %)	6 (60 %)	**<0.001**
Giant cell myocarditis	4 (1.3 %)	2 (0.7 %)	2 (20 %)	**<0.001**
Biomarker levels				
Cardiac troponin, ng/l	691.5 (242.8–1,444.3)	675.0 (230.8–1,363.8)	4,539.0 (560.0–6,104.3)	**0.006**
CK-MB, µg/l	22.4 (8.1–45.7)	21.7 (7.7–45.0)	76.8 (44.1–171.5)	**0.001**
NT-proBNP, ng/l	504.0 (172.5–1,749.5)	453.0 (163.5–1,558.8)	13,850.0 (4,190.0–13,880.0)	**0.003**
CRP, mg/l	32.8 (9.6, 78.1)	32.0 (9.4, 77.3)	66.1 (36.7, 97.3)	**0.030**
Procalcitonin, ng/ml	0.1 (0.1, 0.4)	0.1 (0.1, 0.3)	1.5 (0.8, 6.1)	0.059
Leucocytes, 10^9^/l	9.6 (7.6, 12.5)	9.5 (7.6, 12.0)	17.1 (12.6, 20.9)	**<0.001**
Neutrophils, 10^9^/l	6.5 (4.9, 9.0)	6.4 (4.8, 8.9)	14.3 (8.3, 16.6)	**<0.001**

Numbers are n (percentages), mean (SD) or median (IQR), as appropriate, and compared using Pearson’s Chi-squared test and Wilcoxon rank sum test, respectively. LVEF = left ventricular ejection fraction, MRI = magnetic resonance imaging, CK-MB = creatine kinase muscle and brain, NT-proBNP = N-terminal pro-B-type natriuretic peptide, CRP = C-reactive protein

### 3.2 Severe arrhythmia – Occurrence and characteristics

**Fig. 1:**
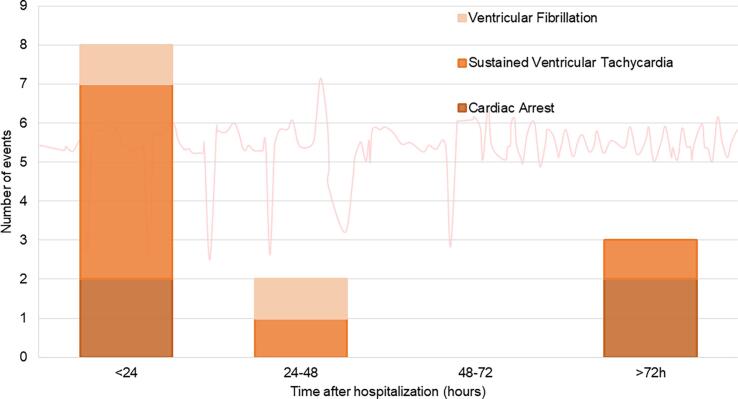
Temporal occurrence of life-threatening arrhythmia during hospitalization. N = 304.

During continuous rhythm monitoring, 13 life-threatening arrhythmias occurred in 10 (3.3 %) patients: 7 sustained VT, 4 CAs, and 2 VF. Life-threatening arrhythmias occurred within 24 h of hospital admission in 62 % and later than 72 h in 23 % of patients (Fig. 1). No life-threatening arrhythmias occurred between 48 and 72 h.

Detailed information on each patient with life-threatening arrhythmia is provided in Table S1. Patients with life-threatening arrhythmia were older (54 vs 41 years, p = 0.017), predominantly female (60 % vs 26 %, p = 0.039), ranked higher in the Killip classification at presentation (p < 0.001), and had a lower median LVEF (30 % vs. 56 %, p < 0.001). Patients with life-threatening arrhythmia also had higher median concentrations of cTnT (4,539 vs 675 ng/l, p = 0.006), NT-proBNP (13,850 vs 453 ng/l, p = 0.003), and CK-MB (76.8 vs 21.7 U/l, p = 0.001). Regarding inflammation, patients with life-threatening arrhythmia had higher median levels of CRP (66.1 vs 32.0 mg/l, p = 0.030), leucocytes (17.1 vs 9.5 10^9^/l, p < 0.001), and neutrophils (14.3 vs 6.4 10^9^/l, p < 0.001). During hospitalization, patients with life-threatening arrhythmia had a significantly higher mortality rate compared to patients without life-threatening arrhythmia (40 % vs. 0.3 %) (p < 0.001) and were more likely to experience cardiogenic shock (70 % vs 4.1 %, p < 0.001). Regarding treatment, patients with life-threatening arrhythmia more frequently received inotropic support (50 % vs 2.4 %, p < 0.001), non-invasive ventilation (20 vs 2.0 %, p < 0.013), and intubation (20 % vs 1.4 %, p < 0.003). There was no difference for beta-blocker use between the two groups (60 % vs 51 %, p = 0.81) (Table S2).

### 3.3 LVEF and troponin T to rule-out life-threatening arrhythmia

**Fig. 2:**
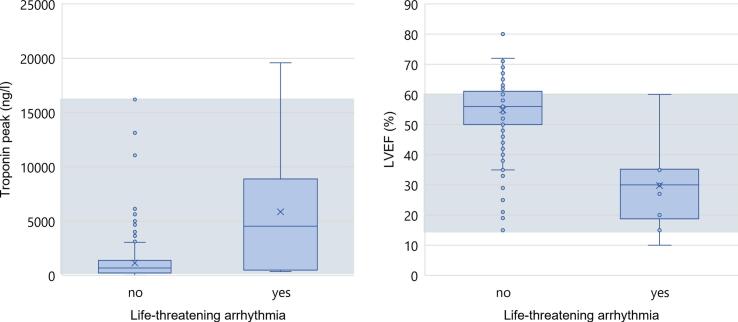
Troponin peak and LVEF values by life-threatening arrhythmia. The rectangular boxes visualize the interquartile range (IQR) spanning from the 25th to the 75th percentile with the central line representing the median. Grey rectangles in the background represent ranges of overlapping values between patients with and without life-threatening arrhythmia. LVEF = left ventricular ejection fraction.

While patients with life-threatening arrhythmia exhibited higher median cTnT levels and lower median LVEF, we did not find a binary cut-off in these two variables to rule-out arrhythmia. We found a substantial overlap in in cTnT levels (67 % of values overlapped) and in LVEF levels (73 % of values overlapped) (Fig. 2).

**Fig. 3:**
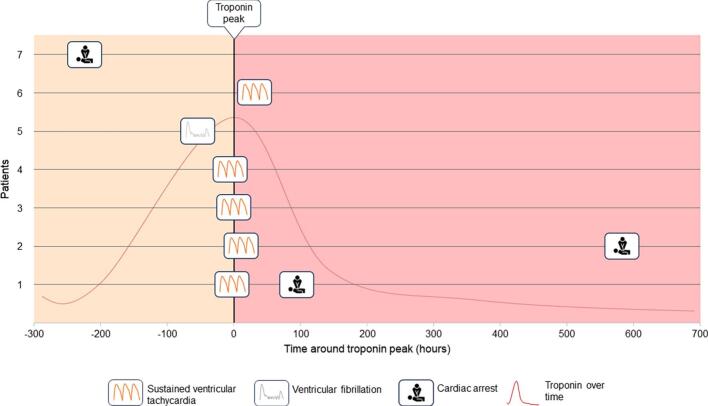
Relationship between troponin peaks and life-threatening arrhythmia. Each bar represents one patient. Types of severe arrhythmias are shown by different shapes. The yellow background represents the time before and the red background the time after the troponin peak. (For interpretation of the references to colour in this figure legend, the reader is referred to the web version of this article.)

In 7 out of 10 patients with life-threatening arrhythmia, at least two hs-cTnT measurements were available, allowing for the definition of a hs-cTnT_peak_. The temporal relationships of life-threatening arrhythmia occurrence and hs-cTnT_peak_ are shown in Fig. 3: 44 % of life-threatening arrhythmias occurred within ± 20 h of the hs-cTnT_peak_. The last life-threatening arrhythmia occurred before the hs-cTnT_peak_ in only 3 (42.9 %) patients, at the same time (±30 min) in 1 patient, and after the peak in 3 (42.9 %) patients. The median times of the last recorded life-threatening arrhythmia before and after the hs-cTnT_peak_ were 54 and 95 h, respectively.

### 3.4 Combined model to rule-out life-threatening arrhythmia

**Fig. 4:**
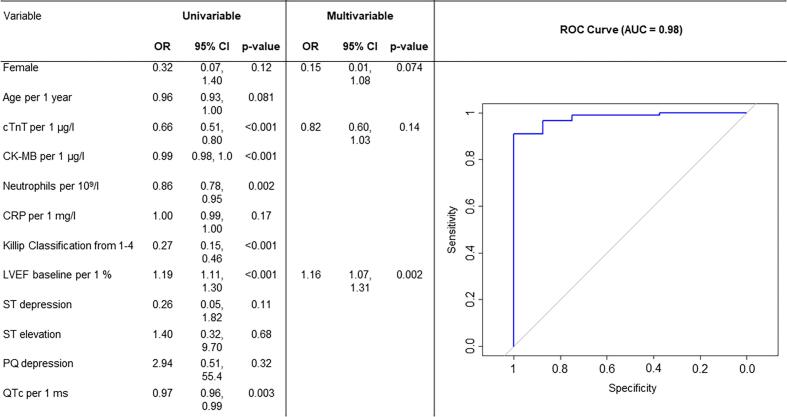
Univariable (left) and multivariable logistic regression model (middle) to rule-out life-threatening arrhythmia. ROC curve (right) for the multivariable logistic regression model (n = 275) to rule-out life-threatening arrhythmia. OR = Odds Ratio, CI = Confidence Interval.

Fig. 4 presents the univariable and the multivariable models to rule-out life-threatening arrhythmia. The selected predictors in the multivariable model were female sex (OR [95 % CI] 0.15 [0.01–1.08], p = 0.074), maximum cTnT levels (OR [95 % CI] 0.82 [0.60–1.03], p = 0.14), and LVEF (OR [95 % CI] 1.16 [1.07–1.31], p = 0.002). The AUC for this combined model was 0.98 (95 % CI 0.96–1), with a sensitivity of 99 % and a specificity of 75 %, translating into a positive predictive value of 99 % for ruling out life-threatening arrhythmia. In sensitivity analyses, we found a similar AUC of 0.98 when we only included patients with available hs-cTnT measurements (Fig. S2).

## 4 Discussion

In a large cohort of patients with suspected AM, our main findings were as follows:

1) Life-threatening arrhythmias were rare, occurring in approximately 1 out of 30 patients with AM, but were associated with a mortality rate of around 40 %.
2) More than half of life-threatening arrhythmias occurred within the first 24 h of hospitalization, but approximately one-fifth only after more than 72 h of monitoring.
3) Patients with life-threatening arrhythmia had higher concentrations of cardiac and inflammatory biomarkers, a lower LVEF, and were more likely to present with heart failure symptoms.
4) There was no binary cut-off value for either LVEF or cTnT levels that could effectively rule out life-threatening arrhythmia. Similarly, surpassing the cTnT_peak_ did not correlate with a lower risk for life-threatening arrhythmia, as half of life-threatening arrhythmias occurred after the cTnT peak.
5) A combined model incorporating female sex, cTnT levels, and LVEF demonstrated a sensitivity of 99 % for ruling out life-threatening arrhythmia in patients with suspected AM, potentially allowing for earlier discharge from rhythm monitoring.

While we observed an overall low risk (3.3 %) of life-threatening arrhythmia in a contemporary patient population with suspected AM, those who did experience life-threatening arrhythmia had a 40 % mortality rate. A fifth of life-threatening arrhythmias occurred after 72 h of monitoring. In the multicenter Lombardy registry, complicated AM − defined by documented, sustained VA, LVEF < 50 % or fulminant presentation − occurred in 27 % of patients with AM and was associated with significantly higher in-hospital mortality and heart transplantation rates [13]. Using nationwide US inpatient survey data, Adegbala et al. reported in-hospital ventricular arrhythmia in about 9 % out of 32,107 patients with AM, with an associated 60 % increase in in-hospital mortality among those who experienced any arrhythmia [18].

Comparing the occurrence of arrhythmias across different studies is challenging due to variations in patient populations, arrhythmia definitions, and sampling methods. The population in this study showed a low risk of life-threatening arrhythmia, which might be due to more conservative definition of of life-threatening arrhythmia with exclusion of non-sustained VT. However, both our findings and those from prior studies highlight a current clinical challenge: more sensitive detection of AM, through the use of high-sensitivity cardiac troponin and modern cardiac MRI, has substantially increased the AM population, leading to lower absolute risks of life-threatening arrhythmia [19]. Recent studies demonstrate the benefit of additional risk stratification through emerging imaging modalities such as T1- and T1- and T2-weighted sequences or late gadolinium enhancement din MRI [[20], [21], [22]]. These techniques may help assess the risk of developing heart failure and dilated cardiomyopathy, and potentially life-threatening arrhythmias. Nonetheless, because of the poor outcomes of patients with life-threatening arrhythmia, a significant proportion of a low-risk, often young patient population with suspected AM undergoes prolonged rhythm monitoring to not miss the rare occurrence of life-threatening arrhythmia. Strategies to identify patients with suspected AM at truly low-risk for life-threatening arrhythmias are therefore needed in order to discharge a large patient population early, that does not need rhythm monitoring.

In our study, we found commonly used clinical variables to be individually insufficient to rule out life-threatening arrhythmia. There was no binary cut-off for LVEF and cTnT levels between patients with and without life-threatening arrhythmia. Similarly, relying solely on the surpassing of the cTnT peak to stop monitoring would have resulted in missing half of life-threatening arrhythmias, despite a clustering of life-threatening arrhythmias around the cTnT_peak_. However, cTnT _peak_data were only available in 7 patients with life-threatening arrhythmia. Of note, previous studies have mainly focused on identifying predictors for the occurrence of ventricular arrhythmia in patients with AM, but not on their exclusion [10,18]. In contrast, we aimed to use clinical variables to rule out life-threatening arrhythmia with a high sensitivity, accepting a more modest specificity. Our final multivariable model included female sex, cTnT levels, and LVEF, and showed excellent sensitivity and positive predictive value for ruling out life-threatening arrhythmia. Applying this model could potentially allow a large proportion of AM patients to be discharged early, thereby reducing the costs associated with continuous monitoring and improving patient comfort. However, before clinical implementation, our model and results require external validation in multicenter and prospective cohorts. This would allow the creation of a model with cutoffs that allow the risk stratification of a specific patient in the clinical setting.

### 4.1 Limitations

While our study benefits from the availability of continuous rhythm monitoring and detailed patient characterization, several limitations must be acknowledged. First, this is a retrospective, observational, single center study, which inherently limits the conclusions that can be drawn and the generalizability. Second, we included not only biopsy-proven AM but also clinically suspected AM with cardiac MRI. While this reflects an AM patient population that physicians typically encounter in clinical practice, where biopsy rates for AM diagnosis are decreasing [23], it might introduce diagnostic variability. Third, due to the low number of patients with life-threatening arrhythmia the statistical power may be limited, and we might encounter potential overfitting of the multivariable model. The model is to be seen as exploratory and needs prospective validation before guiding clinical decisions. Fourth, the cohort predominantly involved young patients from a European country which might limit the applicability to older or more comorbid populations as well as to patients of different ethnicities. We only included patients hospitalized on ICU/IMC limiting the generalizability to patients managed on the general ward with a potential selection bias.

## 5 Conclusion

In patients with suspected AM, life-threatening arrhythmias were rare but associated with a 40 % mortality rate. Commonly used clinical variables performed poorly when used individually to rule out life-threatening arrhythmia. A combined model incorporating readily available clinical variables demonstrated excellent sensitivity to rule-out life-threatening arrhythmia, suggesting its potential use for earlier discharge from rhythm monitoring.

## CRediT authorship contribution statement

**Melina Krempke:** Writing – review & editing, Writing – original draft, Methodology, Formal analysis, Data curation, Conceptualization. **Jasmin Büchel:** Writing – review & editing, Data curation. **Kseniya Bulatova:** Writing – review & editing. **Gianmarco M. Balestra:** Writing – review & editing. **Philip Haaf:** Writing – review & editing. **Jeanne Pouly:** Writing – review & editing. **Paul Drews:** Writing – review & editing. **Christian Mueller:** Writing – review & editing. **Sven Knecht:** Writing – review & editing. **Patrick Badertscher:** Writing – review & editing. **Felix Mahfoud:** Writing – review & editing, Methodology, Conceptualization. **Michael Kühne:** Writing – review & editing. **Christian Sticherling:** Writing – review & editing. **Philipp Krisai:** Writing – review & editing, Supervision, Resources, Project administration, Methodology, Funding acquisition, Conceptualization.

## Declaration of competing interest

The authors declare the following financial interests/personal relationships which may be considered as potential competing interests: **Melina Krempke** has no conflict of interest. **Jasmin Büchel** has no conflict of interest. **Kseniya Bulatova** has no conflict of interest. **Gianmarco M. Balestra** has received speaker honoraria from Inari Medical. **Philip Haaf** has no conflict of interest. **Jeanne Pouly** has no conflict of interest. **Paul Drews** has no conflict of interest. **Christian Mueller** reports receiving research support from the Swiss National Science Foundation, the Swiss Heart Foundation, the University Hospital Basel, the University of Basel; Abbott, Beckman Coulter, Brahms, Idorsia, LSI Medience Corporation, Novartis, Ortho Diagnostics, Quidel, Roche, Siemens, Singulex, Sphingotec, SpinChip, all outside the submitted work, as well as speaker honoraria/consulting honoraria from Amgen, Astra Zeneca, Bayer, Boehringer Ingelheim, BMS, Idorsia, Novartis, Osler, Roche, SpinChip, and Sanofi, all paid to the institution. **Sven Knecht** has received funding of the “Stiftung für kardiovaskuläre Forschung” and the Swiss Heart Foundation. **Patrick Badertscher** has received research funding from the “University of Basel“, the “Stiftung für Herzschrittmacher und Elektrophysiologie”, the “Freiwillige Akademische Gesellschaft Basel”, the “Swiss Heart Foundation” and Johnson&Johnson, and reports personal fees from BMS, Boston Scientific and Abbott, all outside the submitted work. **Felix Mahfoud** is supported by Deutsche Gesellschaft für Kardiologie (DGK), Deutsche Forschungsgemeinschaft (SFB TRR219, Project-ID 322900939), and Deutsche Herzstiftung. Saarland University has received scientific support from Ablative Solutions, Medtronic and ReCor Medical. Until May 2024, FM has received speaker honoraria/consulting fees from Ablative Solutions, Amgen, Astra-Zeneca, Bayer, Boehringer Ingelheim, Inari, Medtronic, Merck, ReCor Medical, Servier, and Terumo. **Michael Kühne** reports grants from the Swiss National Science Foundation (Grant numbers 33CS30_148474, 33CS30_177520, 32473B_176178, 32003B_197524), the Swiss Heart Foundation, the Foundation for Cardiovascular Research Basel and the University of Basel, grants from Bayer, grants from Pfizer, grants from Boston Scientific, grants from BMS, grants from Biotronik, grants and personal fees from Daiichi Sankyo. **Christian Sticherling** has received speaker honoraria from Biosense Webster, Boston Scientific, Biotronik, Microport and Medtronic and research grants from Biosense Webster, and Medtronic. **Philipp Krisai** reports speaker fees from BMS/Pfizer. Grants from the Swiss National Science Foundation, Swiss Heart Foundation, Foundation for Cardiovascular Research Basel, Machaon Foundation.
