# Supplementary material for: Life-threatening arrhythmia in patients with suspected acute myocarditis
Source: Int J Cardiol Heart Vasc. 2025 Jun 19;59:101718. doi: 10.1016/j.ijcha.2025.101718 (PMC12221536; doi:10.1016/j.ijcha.2025.101718)
Supplement: Supplementary Data 1 [file mmc1.docx]

# Supplementary Material

Life-threatening arrhythmia in patients with suspected acute myocarditis

Melina Krempke, MD ^1^, Jasmin Büchel, MD ^1^, Kseniya Bulatova, MSc ^1^, Gianmarco M. Balestra, MD ^1^, Philip Haaf, MD ^1^, Jeanne Pouly, MD ^1^, Paul Drews, BSc ^1^, Christian Mueller, MD ^1^, Sven Knecht, Dsc ^1^, Patrick Badertscher, MD ^1^, Felix Mahfoud, MD ^1^, Michael Kühne, MD ^1^, Christian Sticherling, MD ^1^, Philipp Krisai, MD ^1^

^1^ Department of Cardiology and Cardiovascular Research Institute Basel, University Hospital Basel, University of Basel, Basel, Switzerland

Figures: 2

Tables: 2

Corresponding author: Philipp Krisai, MD

Department of Cardiology

University Hospital Basel

Petersgraben 4, 4031 Basel, CH

philipp.krisai@usb.ch

**Figure S1** summarization of patient flow including the exclusion and inclusion criteria.

**
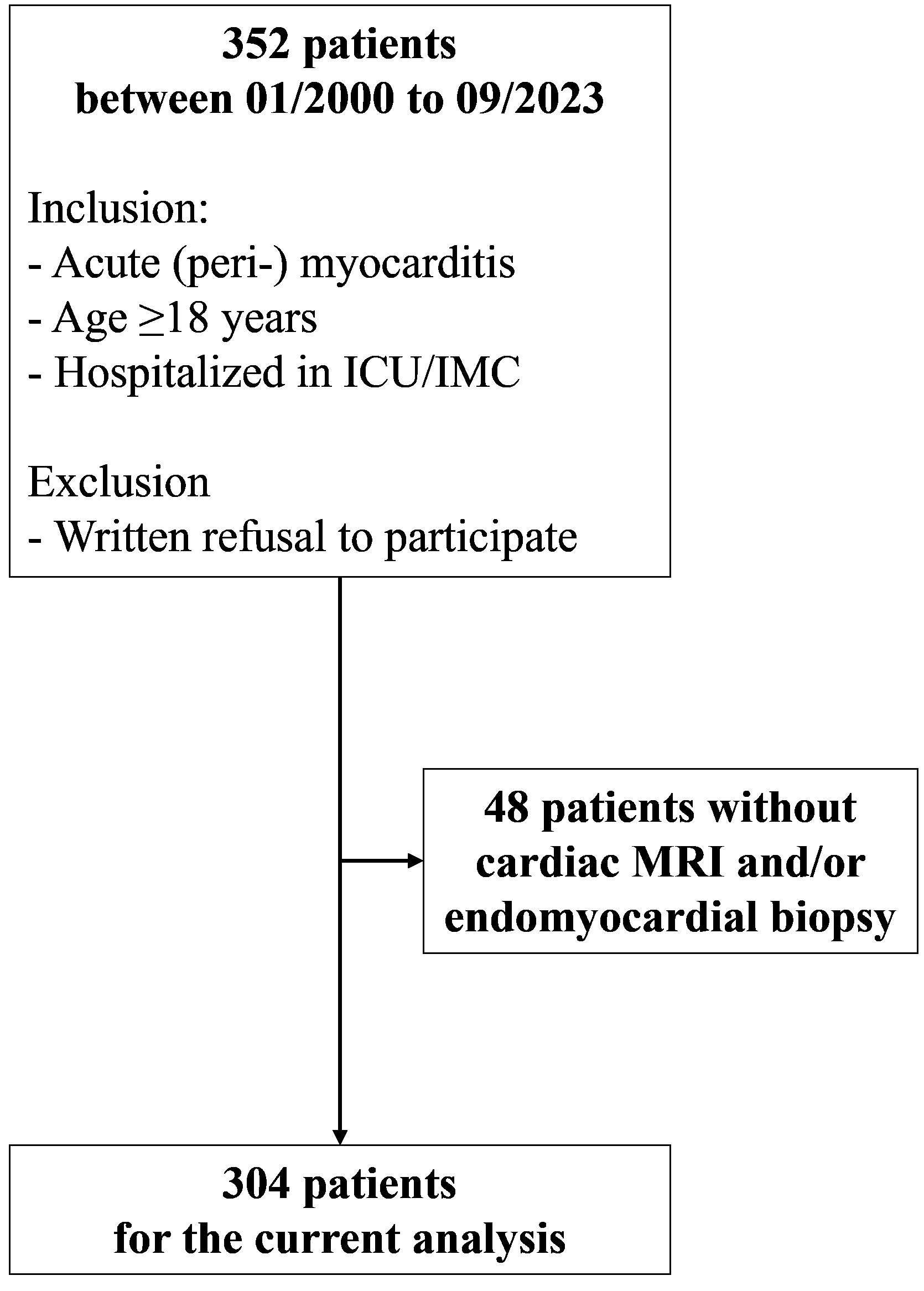
**

**Figure S2** Sensitivity analyses only including patients with available hs-cTnT measurements (n=250). Univariable (left) and multivariable logistic regression model (middle) to rule-out life-threatening arrhythmia. ROC curve (right) for the multivariable logistic regression model to rule-out life-threatening arrhythmia, calculated with hs-TnT. OR = Odds Ratio, CI = Confidence Interval.


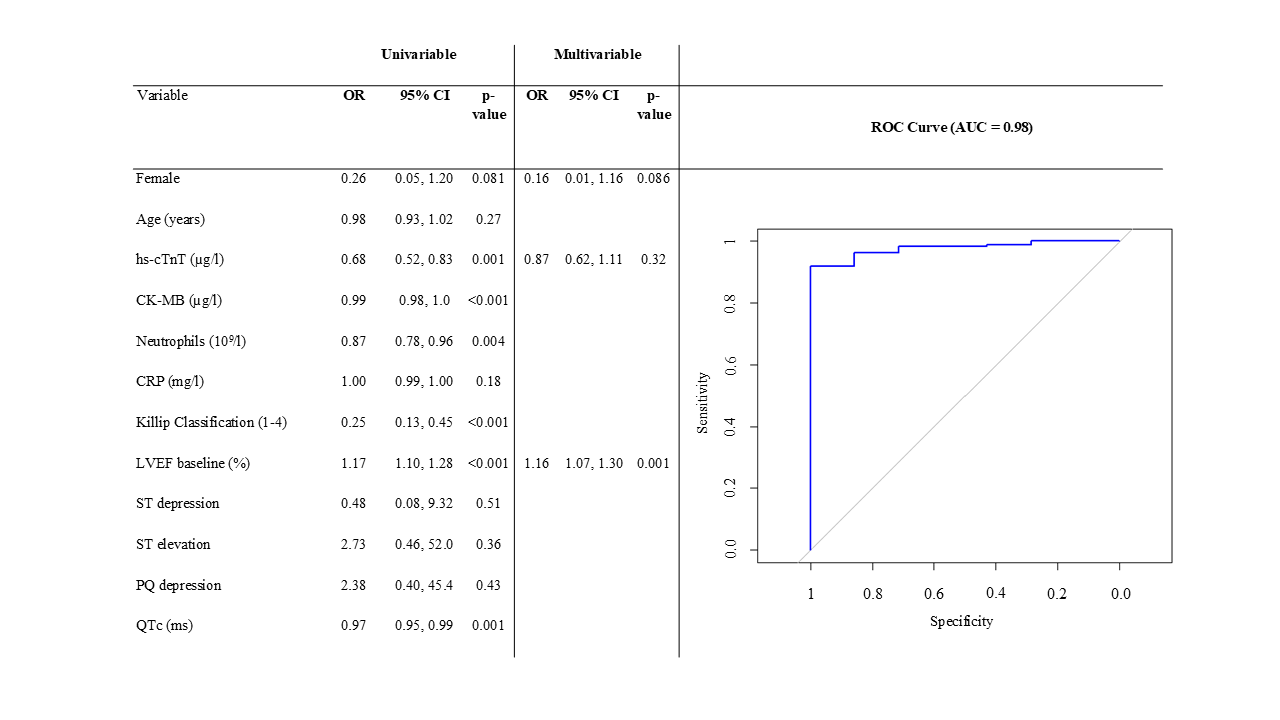


**Table S1** Detailed characteristics of patients with life-threatening arrhythmia.

| **Patient ID** | **Sex** | **Age** | **LVEF (%)** | **Killip classification** | **hs-cTnT (µg/l)** | **CK-MB max (µg/l)** | **Neutrophils max (10^9^/l)** | **CRP max (mg/l)** | **Giant Cell** | **Presenting symptoms** | **12-lead ECG findings** | **Type of life-threatening arrhythmia** | **Occurrence of last life-threatening arrhythmia** |
| --- | --- | --- | --- | --- | --- | --- | --- | --- | --- | --- | --- | --- | --- |
| **1** | m | 54 | 10 | 4 | 19,570 | 146.3 | 24.336 | 220.4 |  | Chest pain, dyspnea | Right Bundle Branch Block, Inverted T Wave | Sustained Ventricular Tachycardia, Cardiac Arrest | >72h |
| **2** | f | 66 | 36 | 1 | 5,010 | 218.8 | 12.566 | 196.8 | Yes | Chest pain, dyspnea, syncope, respiratory infection | AV-Block 1st degree | Sustained Ventricular Tachycardia, Cardiac Arrest | >72h |
| **3** | f | 30 | 30 | 3 | 344 | 22.4 | 8.300 | 43.5 |  | Chest pain, Dyspnea | Right Bundle Branch Block, Left Anterior Fascicular Block, PQ Depression | Sustained Ventricular Tachycardia | <24h |
| **4** | m | 24 | 35 | 4 | 734 | 22.6 | 6.569 | 30.4 |  | Chest pain, Dyspnea, Elevated JVP | Left Bundle Branch Block, Atrial Fibrillation | Sustained Ventricular Tachycardia | <24h |
| **5** | f | 61 | 35 | 1 | 4,068 | 57.2 | 7.680 | 89.0 |  | Dyspnea | Right Bundle Branch Block, Inverted T Wave | Ventricular Fibrillation | 24-48h |
| **6** | m | 52 | 27 | 4 | 5,175 | 44.1 | 16.580 | 100.0 | Yes | Dyspnea | Right Bundle Branch Block, ST Elevation, ST Depression, Inverted T Wave | Sustained Ventricular Tachycardia | 24-48h |
| **7** | f | 47 | 15 | 4 | 6,414 | 294.0 | 18.910 | 36.6 |  | Chest pain | Inverted T Wave | Cardiac Arrest | <24h |
| **8** | f | 65 | 30 | 4 | 502 | 171.5 | 14.295 | 36.8 |  | Syncope | ST Elevation, Atrial Fibrillation | Ventricular Fibrillation, Cardiac Arrest | <24h |
| **9** | f | 61 | 60 | 1 | - | - | - | 33.2 |  | Fever, Respiratory Infection | Right Bundle Branch Block, PQ Depression, ST Derpession | Sustained Ventricular Tachycardia | <24h |
| **10** | m | 77 | 20 | 2 | - | 76.8 | 14.900 | 88.6 |  | Dyspnea, Fever, Pulmonary Crackles, Elevated JVP | Right Bundle Branch Block, ST Elevation, ST Depression, Inverted T Wave | Sustained Ventricular Tachycardia | <24h |

† The patient had continuous rhythm monitoring but there was no 12-lead ECG done.

| **Table S2** In hospital therapy. |  |  | |  |
| --- | --- | --- | --- | --- |
|  | **Overall** | **Life-threatening arrhythmia** | |  |
| **Characteristic** | n = 304 | **no**, n = 294 | **yes**, n = 10 | **p-value** |
| Inotropics | 12 (3.9%) | 7 (2.4%) | 5 (50%) | **<0.001** |
| Mechanical circulatory support | 3 (1.0%) | 2 (0.7%) | 1 (10%) | 0.19 |
| Non-steroidal anti-inflammatory drugs | 209 (69%) | 205 (70%) | 4 (40%) | 0.10 |
| Steroids | 41 (13%) | 36 (12%) | 5 (50%) | **0.003** |
| Colchicine | 15 (4.9%) | 15 (5.1%) | 0 (0%) | >0.99 |
| Intravenous immunoglobulin | 9 (3.0%) | 5 (1.7%) | 4 (40%) | **<0.001** |
| Antiviral therapy | 13 (4.3%) | 10 (3.4%) | 3 (30%) | **<0.001** |
| Cyclosporine | 2 (0.7%) | 1 (0.3%) | 1 (10%) | 0.084 |
| Azathioprin | 1 (0.3%) | 1 (0.3%) | 0 (0%) | >0.99 |
| Diuretics | 51 (17%) | 43 (15%) | 8 (80%) | **<0.001** |
| Beta-Blocker | 156 (51%) | 150 (51%) | 6 (60%) | 0.81 |
| RAAS-Inhibitor | 254 (84%) | 245 (83%) | 9 (90%) | 0.90 |
| Mineralcorticoid-Antagonist | 24 (7.9%) | 22 (7.5%) | 2 (20%) | 0.40 |
| Non-invasive ventilation | 8 (2.6%) | 6 (2.0%) | 2 (20%) | **0.013** |
| Intubation | 6 (2.0%) | 4 (1.4%) | 2 (20%) | **0.003** |
| Numbers are n (percentages) and compared using Pearson’s Chi-squared test, RAAS = Renin-angiotensin-aldosterone system | | | | |
